# The Effect of Plasma Nitriding on the Fatigue Behavior of the Ti-6Al-4V Alloy

**DOI:** 10.3390/ma12030520

**Published:** 2019-02-09

**Authors:** Michele C. B. de Castro, Antônio A. Couto, Gisele F. C. Almeida, Marcos Massi, Nelson B. de Lima, Argemiro da Silva Sobrinho, Mariano Castagnet, Gleicy L. Xavier, Rene R. Oliveira

**Affiliations:** 1 Center for Materials Science and Technology, Instituto de Pesquisas Energéticas e Nucleares, São Paulo 05508-000, Brazil; michelebiondo36@gmail.com (M.C.B.d.C.); acouto@ipen.br (A.A.C.); nblima@ipen.br (N.B.d.L.); mcastag@ipen.br (M.C.); gleicy.limaxavier@gmail.com (G.L.X.); rolivier@ipen.br (R.R.O.); 2 Department of Materials Engineering and Nanotechnology, Universidade Presbiteriana Mackenzie, São Paulo 01302-907, Brazil; marcos.massi@mackenzie.br; 3 Department of Materials and Processes, Instituto Tecnológico de Aeronáutica—ITA/DCTA, São José dos Campos 12228-900, Brazil; argemiro@ita.br

**Keywords:** Ti-6Al-4V alloy, plasma nitriding, fatigue

## Abstract

The Ti-6Al-4V alloy is widely used in the manufacture of components that must have low density and high corrosion resistance and fatigue strength. The fatigue strength can be improved by surface modification. The aim of this study was to determine the influence of plasma nitriding on the fatigue behavior of a Ti-6Al-4V alloy with a lamellar microstructure (Widmanstätten type). Nitriding was executed at 720 °C for 4 h in an atmosphere with N_2_, Ar, and H_2_. Microstructure characterization of the samples was carried out by X-ray diffraction analysis, optical microscopy, and scanning electron microscopy. The average roughness of the specimens was determined, and fatigue tests were executed in a bending–rotating machine with reverse tension cycles (R = −1). X-ray diffraction analysis of the nitrided alloy revealed the following matrix phases: α, β, ε-Ti_2_N, and δ-TiN. A nitrogen diffusion layer was formed between the substrate and the titanium nitrides. Plasma nitriding resulted in an increase in low-cycle fatigue strength, whereas at high cycles of 200 MPa, both conditions exhibited similar behaviors. The fracture surface of the fatigue-tested specimens clearly revealed the lamellar microstructure. The fracture mechanism in the non-nitrided specimens appears to be due to cracking at the interface of the α and β phases of the lamellar microstructure.

## 1. Introduction

Titanium and its alloys are widely used in many areas such as the aerospace, food, naval, and nuclear industries as well as in the production of biomaterials. Among the various titanium alloys, Ti-6Al-4V is the most widely used as it is easy to manufacture and has excellent corrosion resistance, biocompatibility, and a low density. There are certain disadvantages related to the use of this alloy, including low surface hardness, low fatigue strength, low wear properties, and a high friction coefficient [1,2,3,4,5]. Fatigue failure of the Ti-6Al-4V alloy occurs when cyclic stresses are applied to the alloy and the stress levels at which this failure occurs are below the yield stress of the alloy, which is usually determined from tensile tests [6]. This type of failure hinders the use of the Ti-6Al-4V alloy in applications requiring high fatigue strength [7].

Many surface modification techniques can be used to improve the fatigue properties of the Ti-6Al-4V alloy. Among these, the more widely used techniques are heat treatments, coating the surface with films, and thermo-chemical treatments [8]. Nitriding is a thermo-chemical treatment that can be done using gas, laser, or plasma; plasma nitriding is the most efficient because it favors formation of a titanium solid solution with interstitial elements such as nitrogen [8]. Conventional gas nitriding was the preferred technique for a long time. This technique has some disadvantages such as poor surface finish, long duration of the process, and insufficient control over the formation of phases. Currently, plasma nitriding is preferred because it is cheaper, can be done on parts with varied geometries, provides excellent control over the formation of phases, and does not require long processing times to render satisfactory results. One disadvantage of plasma nitriding is the formation of cathode arcs, which could damage the surface of the alloy during treatment [8,9].

Nitriding is done by diffusing nitrogen from the surface into the α phase in the alloy matrix. The diffusion process results in a gradual decrease in nitrogen content from the surface into the alloy, causing hardening. The outermost layer formed during nitriding consists of TiN with a face-centered cubic (FCC) structure. Just below this layer is the Ti_2_N phase with a tetragonal structure. Below these two layers there is a layer of alpha phase titanium (Ti-α) rich in diffused nitrogen [3,5,8]. Formation of the TiN and Ti_2_N phases on the Ti-6Al-4V alloy surface increases the surface hardness and creates compressive stresses on the surface, which delays the crack nucleation stage and thereby increases the fatigue life of the alloy [10,11].

Plasma nitriding can be affected by many factors, but the main ones are the duration of nitriding, temperature, and the gas composition in the nitriding atmosphere. A number of studies have been carried out to compare the thickness of nitride layers as a function of process time and temperature, and further, to determine the effect of the nitride layer thickness on improvements in fatigue properties. According to Rahman et al. [12] in their studies on plasma nitriding, there was a marked improvement in fatigue properties when the treatment was done at 500 °C for 6 h in a nitriding atmosphere containing nitrogen and hydrogen in the proportion 3:1. Rodriguez et al. [10] carried out nitriding at 850 °C and 900 °C for 1 to 4 h and reported that the nitride layer had a positive effect on the fatigue properties. This effect was caused by the compressive residual stress in the alloy, which delayed the crack nucleation step. According to Farokhzadeh et al. [1], plasma nitriding carried out at 600 °C for 24 h revealed a 23% improvement in fatigue strength of the Ti-6Al-4V alloy, compared to a non-nitrided alloy. In a previous work carried out by our research group, Almeida et al. [13] performed plasma nitrided tests in creep specimens of the Ti-6Al-4V alloy, where a variety of nitriding parameters were tested. In this present work, the same nitriding conditions [13] were used, with small adjustments due to the change of geometry of the specimen.

By varying the microstructure and nitriding parameters, the fatigue strength of a material can be affected. Tokaji et al. [14] reported that the fatigue strength of pure titanium was increased by nitriding, while it was decreased in the case of Ti-6Al-4V. Based on detailed observations of fatigue crack initiation, growth, and fracture surfaces, the improvement and the reduction in fatigue strength by nitriding of pure titanium and Ti-6Al-4V alloys were primarily attributed to enhanced crack initiation resistance and to premature crack initiation of the nitride layer, respectively. However, Farokhzadeh et al. [1] achieved an improvement on the fatigue proprieties of the Ti-6Al-4V alloy plasma nitrided at a low temperature (600 °C). These results were different than those obtained by an elevated temperature treatment (900 °C) as well as conventional gas/plasma nitriding treatments reported in the literature [14,15,16]. The thin morphology of the compound layer restricted the extent of premature crack initiation from the surface. Moreover, a deep diffusion zone with a well-bonded interface decreased the likelihood of fatigue initiation at (or below) the compound layer interface [1].

In view of these observations, the objective of this study was to evaluate the fatigue behavior of the Ti-6Al-4V alloy before and after plasma nitriding. The starting Ti-6Al-4V alloy had a Widmanstätten-type microstructure. After the plasma nitriding, the nitride layers were studied, and fatigue tests were carried out in a bending–rotating machine.

## 2. Materials and Methods

In this study, a Ti-6Al-4V alloy with composition conforming to the ASTM F136-13 [17] standard was used. The alloy was acquired in the form of annealed rods 12.7 mm in diameter and 3140 mm long. Specimens for the fatigue and the tensile tests were machined from the Ti-6Al-4V rods as per the ASTM E8-11 [18] standard. The specimens used for the fatigue and the tensile tests were heat treated to obtain a Widmanstätten-type microstructure. The heat treatment was carried out at 1050 °C for 30 min in an argon atmosphere. The heating rate was 20 °C/min and the specimens were subsequently cooled at 6 °C/min.

The heat-treated specimens were ultrasonically cleaned in a mixture of water with a surfactant and then cleaned in isopropyl alcohol for more than 15 min before plasma nitriding. Plasma nitriding was carried out in a mixed gas flux containing N_2_, Ar, and H_2_ in the proportion 5:5:1. Almeida et al. tested [13] some nitriding conditions for the same material, and those previously defined conditions were used in this work, with small adjustments due to the change of geometry of the specimen. Table 1 shows the average process parameters that were used along with their standard deviations.

Microstructure characterization of as non-nitrided and nitrided Ti-6Al-4V alloy samples was carried out using optical microscopy, scanning electron microscopy, and X-ray diffraction analysis. Initially, 5-mm-thick cross-sectional samples were cut from the rods. These samples were mounted and ground sequentially with SiC papers of 280, 400, 600, and 1200 mesh. Subsequently, the samples were polished with colloidal silicon containing 0.04-μm particles. The etchant used to reveal the microstructure was a solution of 10 mL hydrofluoric acid (HF) and 2.5 mL nitric acid (HNO_3_) for 3 s. The etched samples were examined in an Olympus BX60 optical microscope (Olympus Corporation, Tokyo, Japan). The non-nitrided and nitrided samples were also studied by X-ray diffraction analysis using Cu kα (λ = 0.1542 nm). The specimens were scanned at angles between 20° to 80° using a scan rate of 2° min^−1^. Evaluation of the X-ray diffractograms was done with the software Crystallographica Search-Match 3.1.0.2 (Oxford Cryosystems, Oxford, UK).

The surface roughness of the non-nitrided and nitrided samples was determined. This was done with a Mitutoyo roughness measuring device (Mitutoyo Corporation, Sakado, Japan). In these measurements, a sample cutoff length of 0.08 mm was used, as per the ISO 4287:1997 [19] standard. Five random measurements were carried out on every sample and then the averages and standard deviations of the measured values were determined. The roughness parameter used was Arithmetic Roughness (Ra). The tensile tests were carried out as per the ASTM E8-11 [18] standard in a universal mechanical testing machine Instron 4400R (Instron, Norwood, MA, USA) with the crosshead speed of 1 mm/min.

The fatigue tests were carried out in a bending-rotating machine with reverse tension cycles. Therefore, in the fatigue tests on untreated and nitrided specimens, a loading ratio of R = −1 and rotation velocity of 3000 rpm were used. At least three specimens in each condition (non-nitrided and nitrided) were tested at each applied stress cycle. The fatigue tests were started at stress cycles equal to 60% of the yield stress. In subsequent tests, the stresses were lowered until they reached a point at which the specimen did not fail even after about 10^7^ cycles. The stress versus the number of cycles (S–N) curves were plotted from the medium values of the fatigue test results. The fracture surfaces of the specimens that failed in the fatigue tests were examined in a Philips XL-30 scanning electron microscope (Philips, Eindhoven, The Nederlands).

## 3. Results and Discussions

**Figure 1:**
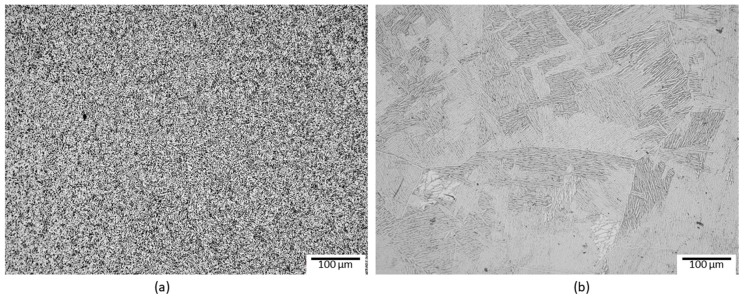
The Ti-6Al-4V alloy in the as-received annealed condition with equiaxial microstructure (**a**) and after heat treatment to obtain the Widmanstätten-type microstructure (**b**).

Figure 1a shows the equiaxial type microstructure of the Ti-6Al-4V alloy in the as-received state (annealed at 800 °C/2 h). The matrix consists of uniformly distributed α phase grains (light regions) and β phase grains (dark regions). Figure 1b shows the microstructure of the alloy after heat treatment at 1050 °C for 30 min. In this micrograph, a Widmanstätten-type microstructure can be observed with α and β phases in the lamellas form.

**Figure 2:**
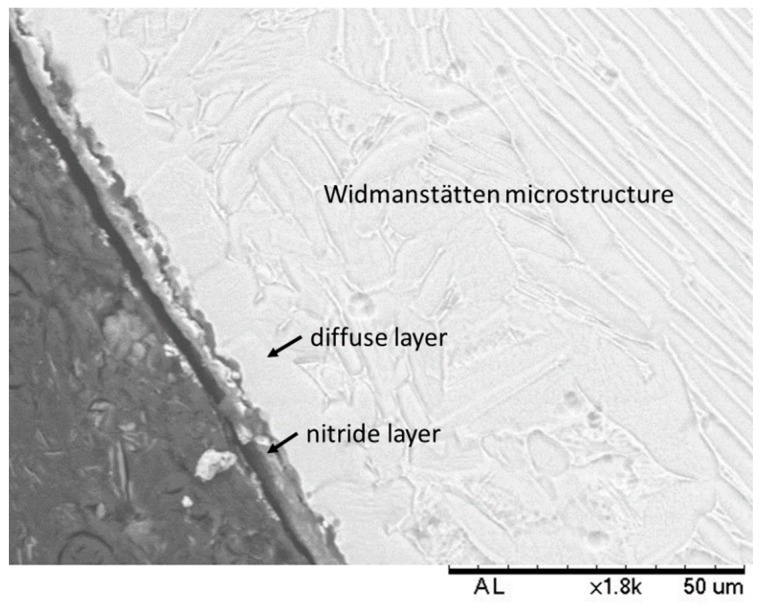
Scanning electron micrograph of the Ti-6Al-4V alloy after plasma nitriding. The arrows indicate the titanium nitride layer and the nitrogen diffusion layer in the matrix.

In the scanning electron micrograph shown in Figure 2, the two stages of formation of the nitride layer can be observed. The first stage forms by the interaction between the alloy and the active particles in the nitriding atmosphere, and the second stage forms by diffusion of nitrogen atoms into the bulk of the Ti-6Al-4V alloy [3]. Nitriding of this alloy results in two layers. The first layer is close to the surface and consists of titanium nitrides (Ti_2_N and TiN), and right below this layer is a region in which nitrogen atoms have diffused in the α phase of the Ti-6Al-4V alloy. Below these two layers, the matrix with a Widmanstätten-type microstructure can be observed. It is also possible to observe that the layers have varying thicknesses, which makes it difficult to measure the thicknesses of the layers. Nevertheless, the thickness of the nitride layer was 3.77 ± 1.46 µm and that of the diffuse layer was 10.37 ± 2.58 µm.

Zimmer et al. [20] reported that the nitride layer could have thicknesses varying from 1 to 50 µm and that the diffuse layer could attain thicknesses of hundreds of micrometers. According to Yildiz et al. [3], the nitride layer thickness was up to 2 µm when the alloy was plasma nitrided at an average temperature of 700 °C. As per Farokhzadeh et al. [1], when samples were nitrided at 600 °C, the thickness of the nitride and diffuse layers were 2 and 44 µm, respectively; in samples nitrided at 900 °C, the thicknesses of the nitride and diffuse layers were 5.8 and 19 µm respectively. Rahman et al. [12] reported that the thickness of the nitride layer was 3 µm when the alloy was nitrided at 700 °C and 9 µm when nitrided at 900 °C.

The thicknesses of the nitride layers in this study are consistent with those reported by other authors [1,3,8,12]. The thicknesses of the layers in the nitrided samples are mainly influenced by the plasma-nitriding-process parameters such as temperature (primarily), duration, and atmosphere, among others [8]. Increase in process temperature increases the nitrogen diffusion rate and this increases the thickness of both layers [12]. Rodriguez et al [10] reported that the influence of the layers formed upon plasma nitriding could be beneficial for the alloy because the residual stress on the surface delays cracks nucleation, increasing the fatigue strength of the alloy.

The arithmetic surface roughness of the non-nitrided specimens was 0.38 ± 0.13 µm and that of the nitrided specimens was 0.62 ± 0.12 µm. Plasma nitriding significantly increased the roughness of the specimens (more than 50%). This result was foreseen and was due to ion bombardment of the alloy surface during plasma nitriding. Rahman et al. [12] and Yildiz et al. [3] reported that ion bombardment of the alloy leads to an increase of the surface roughness. The higher the temperature and duration of plasma nitriding, the higher is the extent of the bombardment of the surface and, consequently, the higher the roughness. Roughness, like hardness and layer thickness, increases with an increase in temperature and duration of treatment [1,3,12].

**Figure 3:**
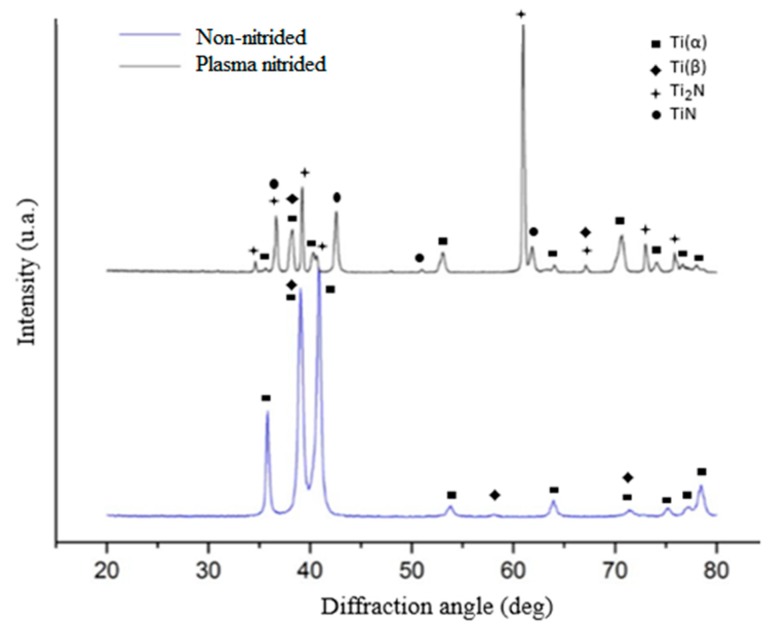
X-ray diffraction of the non-nitrided and nitrided Ti-6Al-4V alloy. Top: plasma nitrided alloy. Bottom: non-nitrided alloy.

The X-ray diffraction results of the non-nitrided and nitrided Ti-6Al-4V alloy are shown in Figure 3. In the non-nitrided alloy, only two phases could be identified: α phase (JCPDS 44-1294) with hexagonal close-packed (HCP) structure and β phase (JCPDS 44-1288) with body-centered cubic (BCC) structure; these observations are in agreement with other authors [2,3]. In the untreated alloy, three peaks corresponding to reflections from the planes (110), (200), and (211) of the β phase can be observed. However, two of these are coincident with peaks corresponding to reflections from the planes (002) and (103) of the α phase. The only peak of the β phase that does not coincide with peaks of the α phase is the peak corresponding to the reflection from the plane (200), but with low relative intensity. The β phase peak with the highest intensity is that related to the plane (110), and this coincides with the reflection from the plane (002) of the α phase. The coincidence of peaks makes it difficult to identify the β phase in the Ti alloys using only X-ray diffraction when the α phase is also present [13].

X-ray diffraction analysis of the nitrided alloy (Figure 3) revealed four phases: δ-TiN (JCPDS 38-1420) with face-centered cubic (FCC) structure, ε-Ti_2_N (JCPDS 17-0386) with a tetragonal structure, and the α and β phases that were seen in the untreated specimen. These observations are in agreement with previous studies [2,3,8,10]. Some peaks of α and β phases disappeared due to the smaller thickness of the substrate (Ti-6Al-4V alloy) analyzed when compared to the layer thickness of the nitrites formed. Alteration in the position of the diffraction peaks of the α phase was also noted. This was due to the dissolution of nitrogen to form an interstitial solid solution, causing the expansion of crystal lattice of the α phase. Similar observations were reported previously [2,3]. Yetim et al. [2] and Yildiz et al. [3] also observed that the peak intensity of the Ti_2_N phase decreased with the increase in plasma nitriding time and with the increase in temperature. Depending on the thickness of the nitride layer, these peaks tended to disappear. This is caused by the increase in diffusion rate of nitrogen in the α phase, resulting in a thicker diffuse layer and formation of only the TiN phase on the surface. In general, Ti_2_N is the main phase formed in low-temperature nitriding and TiN in high-temperature nitriding [2].

Tensile tests were carried out on untreated specimens and their yield stress (σ_e_), ultimate tensile stress (σ_max_), and elongation were determined. These tensile tests were carried out to aid in the determination of the parameters for the fatigue tests. The yield stress, ultimate tensile stress, and elongation were 1017.56 ± 7.92 MPa, 1076.36 ± 8.56 MPa, and 16.64 ± 0.47%, respectively. These results are similar to those reported in other studies [1,5,12].

**Figure 4:**
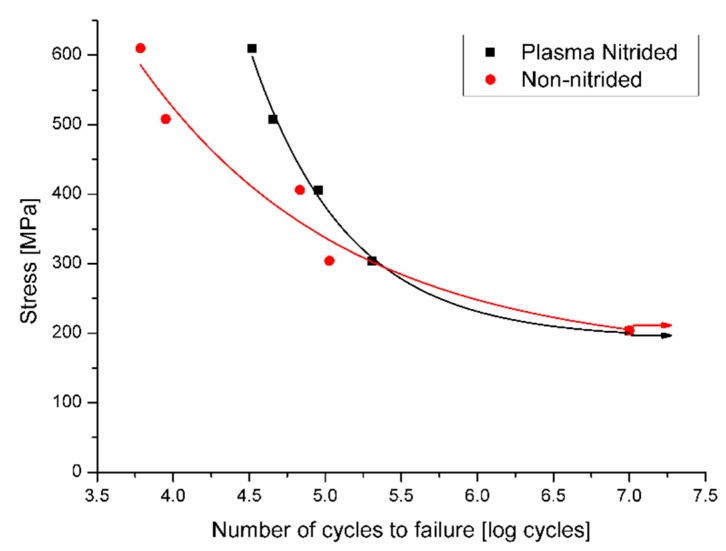
Graphic of stress versus number of cycles obtained from the fatigue tests on the non-nitrided and nitrided Ti-6Al-4V alloy. Arrows indicate samples that did not fail.

Figure 4 shows the graph S–N curves of the non-nitrided and plasma-nitrided Ti-6Al-4V alloy. At relatively high-stress amplitudes (low cycle fatigue region), this graph shows that the nitrided specimens failed. Overall, in low amplitude stress tests (high cycle fatigue region), no failures occurred until interruption of the tests after 10^7^ cycles (run-out). In general, it can be observed that the nitrided specimens withstood a higher number of cycles than the non-nitrided specimens before failure at higher stress amplitudes and number of cycles lower than 10^5^ cycles. In similar fatigue studies, the same behavior was also observed by Zuo et al. [7]. At high-stress levels, the nitrided specimens withstood a higher number of cycles to failure. At low-stress levels, the behavior of nitrided and non-nitrided conditions were similar.

Increased fatigue life of the plasma nitrided specimens in tests at higher stresses can be attributed to compressive residual stress in the nitride layers and in the nitrogen diffusion zone, thereby inhibiting crack nucleation at the alloy surface. This is in agreement with another study by Rodriguez et al. [10]. According to Farokhzadeh et al. [1], the nitride layer reduces the extent of brittle failure on the specimen surface while the diffusion zone provides mechanical support.

As per the study of Morita et al [21], the formation of these layers after plasma nitriding increases the surface hardness of the alloy, which in turn increases the resistance to movement of dislocations and, as a consequence, increases the fatigue strength of the alloy. Rocha [22], reported that delay in crack nucleation in plasma nitrided specimens is due to the difference in the Young modulus between the titanium alloy matrix (100 GPa) and the titanium nitrides formed on the plasma nitride surface (400 GPa). When high stresses are applied to the fragile nitride layer, it causes the nucleation and the growth of a crack in this layer. At lower applied stresses, the nitride layer has no significant effect on improving fatigue behavior. This seems to be related to an increase in surface roughness during plasma nitriding, and this plays a more important role than nitriding itself on the fatigue life of Ti-6Al-4V alloy specimens. The effect of the increase in surface roughness on the mechanical behavior of the plasma nitride Ti-6Al-4V alloy has also been reported by other authors [1,23,24,25].

**Figure 5:**
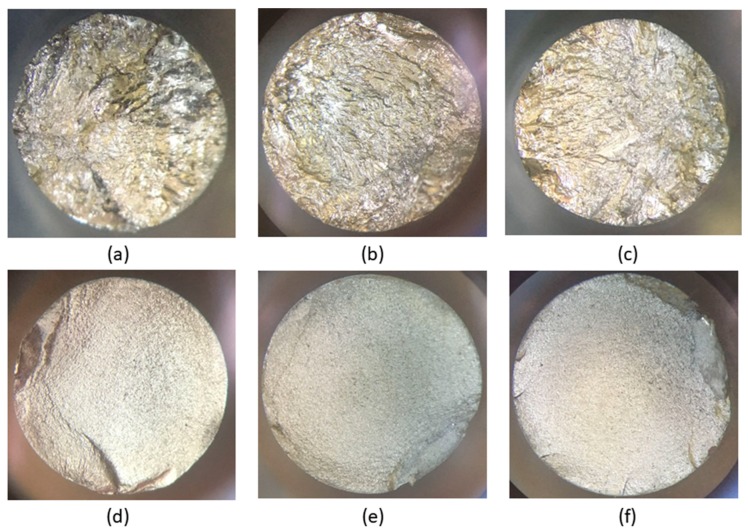
Fracture surfaces of specimens after the tensile test. These images were obtained using a stereoscopic microscope. Conditions: (**a**) non-nitrided specimen tested at 304 MPa and 123,700 cycles to failure, (**b**) non-nitrided specimen tested at 406 MPa and 68,700 cycles to failure, (**c**) non-nitrided specimen tested at 610 MPa and 6700 cycles to failure, (**d**) nitrided specimen tested at 304 MPa and 233,000 cycles to failure, (**e**) nitrided specimen tested at 406 MPa and 90,800 cycles to failure, (**f**) nitrided specimen tested at 610 MPa and 35,000 cycles to failure.

To observe the fracture surface of the fatigue tested specimens, three loads were selected: 304, 406, and 610 MPa. At each selected load, the fracture surfaces of two specimens in the non-nitrided and nitrided conditions were observed. Figure 5 shows the general features on the fracture surfaces of the specimens. It can be seen that at low magnifications, the fracture surfaces of the nitrided specimens (Figure 5d–f) are quite different from those of the non-nitrided specimens (Figure 5a–c).

The non-nitrided specimens reveal a ductile-type fracture. The surface in relief is characteristic of plastic deformation prior to fracture. The fracture surfaces of the nitrided specimens that were fatigue tested reveal a large region of planar propagation, followed by a relatively small region corresponding to final failure. This has also been reported by other authors [1,10]. On this surface, it is difficult to determine the region corresponding to the initial stages of the crack. Similar aspects about fracture surface features of nitrided specimens that were fatigue tested were reported by other authors [1,7]. One feature to highlight about the fracture surfaces of nitrided specimens that were fatigue tested is the number of points at which cracks initiated. When low stresses were applied, a single crack initiation point was observed, as presented in Figure 5b, whereas at high stresses, there were many crack initiation points, characterized by ratchet marks from the nucleated cracks, as presented in Figure 5f. The ratchet marks are indicative of multiple crack fronts that rapidly progress from the surface into the bulk of the specimen, leading to its premature failure. These observations are in agreement with those made by Cassar et al. [25].

**Figure 6:**
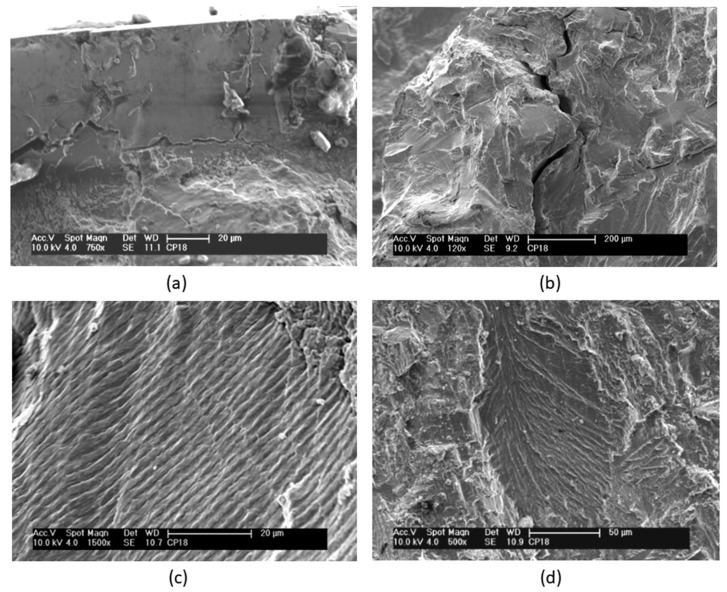
Scanning electron micrographs of the fracture surfaces of nitrided specimens after fatigue tests at 304 MPa and 233,000 cycles to failure. (**a**) A planar region where the crack originated. (**b**) Microcracks perpendicular to the propagation direction. (**c**) Striations. (**d**) Lamellar microstructure consisting of α and β phases.

Details of the fracture surfaces of the nitrided specimens that were fatigue tested are shown in Figure 6. No significant differences in the fracture surfaces were observed in the fatigue specimens tested at different stress amplitudes. Figure 6a shows a planar region where the crack originated. On this fracture surface, sub-cracks perpendicular to the fracture surface can also be seen. Overall, extensive plastic deformation can be observed throughout the fracture surface, without evidence of brittle fracture.

**Figure 7:**
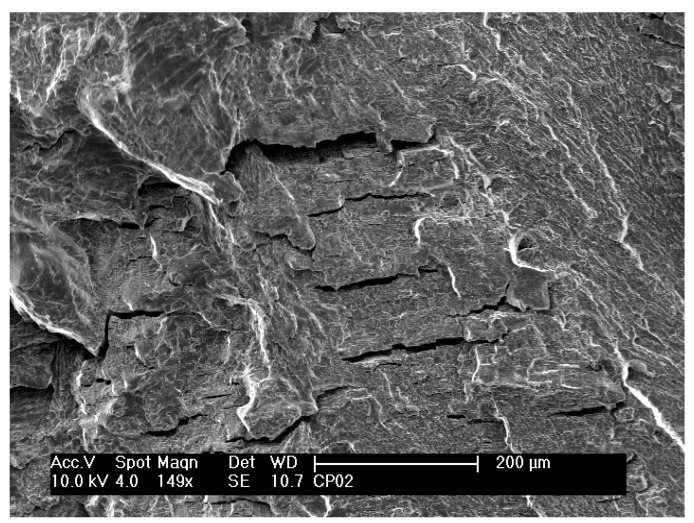
Scanning electron micrograph of the fracture surface of non-nitrided specimen after a fatigue test at 610 MPa and 6700 cycles to failure.

Over the whole fracture surface, microcracks and striations perpendicular to the propagation direction can be seen in detail in Figure 6b,c, respectively. Multiple cracks were also reported in other studies [1,21,26]. A large number of striations on the fracture surface indicate plastic deformation. At various regions on this fracture surface, a characteristic lamellar microstructure consisting of α and β phases can also be clearly observed in Figure 6d. Planar facets, which have the same size and morphology as the lamellas or a colony of lamellas, typical of lamellar microstructures (Widmanstätten type), can also be observed. Similar observations were also reported in other studies [1,26]. Microcracks seem to occur at the interfaces of the α and β phases of the lamellar microstructure [7]. The fractography in Figure 7 shows the formation of fracture cracks at the lamella interfaces.

**Figure 8:**
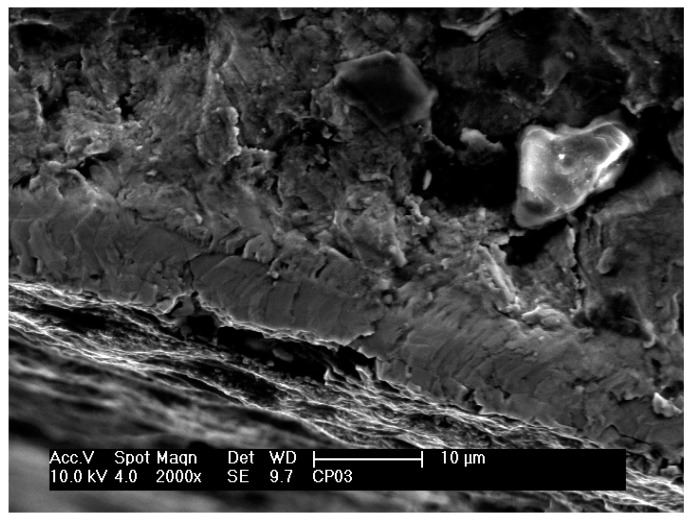
Scanning electron micrograph of the fracture surface of the plasma nitrided specimen that was fatigue tested at 610 MPa and 233,000 cycles to failure.

**Figure 9:**
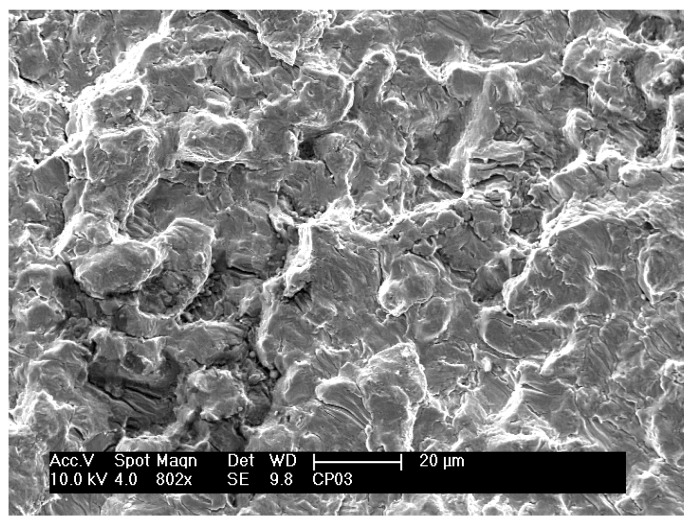
Scanning electron micrograph of the fracture surface of the plasma nitrided specimen that was fatigue tested at 610 MPa and 233,000 cycles to failure.

**Figure 10:**
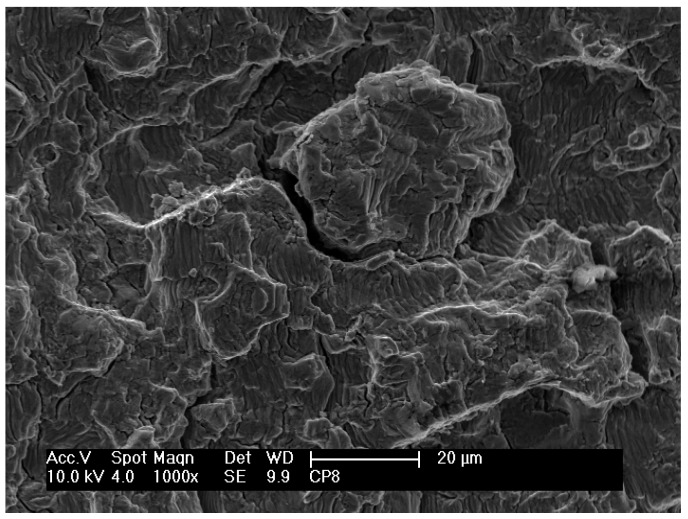
Scanning electron micrograph of the fracture surface of the plasma nitrided specimen that was fatigue tested at 406 MPa and 90,800 cycles to failure.

**Figure 11:**
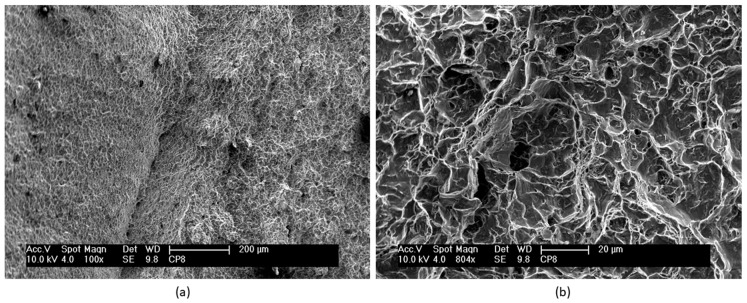
Scanning electron micrographs of the final rupture region on the fracture surface of the plasma nitrided specimen that was fatigue tested at 610 MPa and 90,800 cycles to failure. (**a**) Dimples. (**b**) Detail of the dimples.

Details of the fracture surface of nitrided specimens that were fatigue tested are shown in Figure 8, Figure 9, Figure 10 and Figure 11. Figure 8 shows a detail of the nitride layer region after fatigue testing at 610 MPa and after 35,000 cycles to failure. A region at the border of the nitrided specimen that was fatigue tested shows the same features. The nitrides layer cannot be observed, but only the nitrogen diffusion layer. It can be noted that the thickness of this layer is irregular throughout the surface of the specimen. The presence of striations along the whole layer can also be observed. Striations in the nitride layer were also observed by Farokhzadeh et al. [1].

Figure 9 shows a region close to an initiated crack on the fracture surface of a nitrided specimen that was fatigue tested at 610 MPa and 35,000 cycles to failure. These observations are similar to those observed in nitrided specimens that were fatigue tested at other stress cycles. In this region, planar facets and microstructural characteristics of the same size and morphology of a lamella or a colony of lamellas can be observed, similar to those observed on fractured untreated specimens. As reported in other studies [7,27], fatigue crack initiation and propagation in lamellar materials depends on plastic anisotropy of colonies and cleavage fracture. Shear processes parallel to lamellar interfaces are relatively easier than across the lamella. Thus, easy propagation of cracks parallel to lamellar interfaces results in facets on the fracture surface. In contrast, it is possible to observe in Figure 10 on the fracture surface of another specimen (406 MPa and 90,800 cycles to failure) the layers of fractured lamellas caused by microcracks at the α–β interfaces.

Zuo et al. [7] also observed that microcracks occurred more frequently at the α–β interfaces. According to Zuo et al. [7], in a material with two phases in its microstructure (α and β), the crack nucleation region depends not only on microstructural aspects but also on the response of each phase to the stress applied during the test. When stress is applied to the alloy, the response to this stress differs depending on the phase. The β phase being more ductile than the alpha phase permits dislocation sliding to occur. Dislocation sliding continues until it reaches the α–β interface. At this interface, sliding ceases and stress accumulates. Since the alpha phase is less ductile, the accumulated stress causes cracks to nucleate. As a result, a number of α–β interfaces become spots for crack initiation.

The final rupture region on the fracture surface of a nitrided specimen that was fatigue tested at 406 MPa and 90,800 cycles to failure is shown in Figure 11. In Figure 11a, note the microcavities (dimples) all over the surface, characteristic of a final rupture region caused by mechanical overload. Figure 11b shows Figure 11a at a higher magnification and this reveals in detail the size and morphology of the microcavities (dimples). The microcavities (dimples) are of varying sizes and are predominantly large and shallow, typical of shearing (shear dimples). Throughout the final rupture region, no oval microcavities, typical of tearing (tear dimples), were observed.

## 4. Conclusions

This study about the effect of plasma nitriding on the fatigue behavior of the Ti-6Al-4V alloy enabled us to make the following conclusions:

- Thermochemical treatment by plasma nitriding resulted in the formation of two nitride layers: δ-TiN with a face-centered cubic structure and ε-Ti_2_N with a tetragonal structure. Below these layers, a region where nitrogen had diffused in the alloy was encountered. The thicknesses of the nitride layer and the nitrogen diffusion layer, as measured by scanning electron microscopy, were 3.77 ± 1.46 μm and 10.37 ± 2.58 μm, respectively, for the particular parameters adopted in the nitriding process.
- The plasma nitrided specimens had higher low-cycle fatigue strength, while at high cycles, the nitrided and non-nitrided specimens exhibited similar behavior. There was a increase in surface roughness of the alloy caused by the plasma nitriding process (from 0.38 to 0.62 µm). This increase in roughness seems to cancel the positive effect of nitriding on fatigue properties at high cycles.
- In the untreated specimens that were fatigue tested, a fracture surface with raised regions, characteristic of plastic deformation prior to fracture, was observed. The fracture surfaces of the nitrided specimens revealed large regions with planar propagation, followed by a relatively smaller region corresponding to final rupture.
- At various regions on the fracture surfaces of specimens that were fatigue tested, a lamellar microstructure was clearly observed. The fracture mechanism in the non-nitrided specimens seems to be cracking at interfaces of the α and β phases in the lamellar microstructure.
- The fracture surface at the edges of the nitrided specimens that were fatigue tested revealed a diffusion layer with a varying thickness and the presence of striations throughout the whole layer. The final fracture region showed a rupture characteristic of mechanical overload with a large number of shear dimples.

## Figures and Tables

**Table 1:** Average and standard deviations of parameters used in plasma nitriding.

Temperature (°C)	Pressure (Pa)	Current (A)	Voltage (V)	Power (W)
730 ± 3	586.62	2.65 ± 0.04	416 ± 1	1102 ± 24
